# Blood–brain barrier integrity disruption linked to brain lesions and relapse prediction in Southern Chinese anti-NMDAR encephalitis patients

**DOI:** 10.3389/fncel.2026.1872425

**Published:** 2026-07-06

**Authors:** Lingling Tong, Hui Chen, Yanzhen Huang, Wenlin Jiang, Wen Huang

**Affiliations:** Department of Neurology, The First Affiliated Hospital of Guangxi Medical University, Nanning, Guangxi, China

**Keywords:** autoimmune encephalitis, blood–brain barrier, brain lesion, oligoclonal bands, prognosis

## Abstract

**Objective:**

This study aims to investigate the characteristic brain abnormalities in Southern Chinese patients with anti-N-methyl-D-aspartate receptor (NMDAR) encephalitis and to explore their association with blood–brain barrier (BBB) disruption and clinical implications.

**Methods:**

This study retrospectively analyzed 80 cases of anti-NMDAR encephalitis. Patients were divided into a group with abnormal brain MRI (*n* = 35) and a group with normal brain MRI (*n* = 45). The extent of BBB disruption was assessed using the albumin quotient (Qalb), defined as the cerebrospinal fluid (CSF)-to-serum albumin ratio. Disease severity was evaluated using the modified Rankin Scale (mRS). Patients were further categorized into a relapse group and a non-relapse group based on post-discharge relapse status. Key predictive factors were identified through multivariate regression analysis.

**Results:**

Brain MRI abnormalities were observed in 43.75% (35/80) of the anti-NMDAR encephalitis patients. Among these, typical MRI findings of anti-NMDAR encephalitis abnormalities were present in 26 patients (32.5% of the total cohort). Brain MRI abnormalities were significantly associated with abnormal BBB permeability (42.9% vs. 13.3%, *p* = 0.003). Multivariate logistic regression analysis identified two independent predictive factors for brain MRI abnormalities: BBB dysfunction (OR = 4.088, 95% CI: 1.031–16.213, *p* = 0.045) and time from symptom onset to first-line immunotherapy (OR = 1.041, 95% CI: 1.001–1.083, *p* = 0.045). Furthermore, receiver operating characteristic (ROC) curve analysis demonstrated that the combined model of BBB dysfunction and time from symptom onset to first-line immunotherapy exhibited favorable predictive performance, with an area under the curve (AUC) of 0.745 (95% CI: 0.634–0.857). BBB dysfunction was closely associated with multi-regional cerebral involvement (*p* = 0.002). Further Spearman correlation analysis showed that impaired BBB integrity correlated with greater MRI lesion severity and broader lesional distribution (*p* = 0.003). In binary logistic regression analysis adjusting for mRS score, positive oligoclonal bands (OCBs) were identified as a potential predictor of relapse, with an OR of 7.991 (95% CI: 1.242–51.411, *p* = 0.029). The AUC was 0.730 (95% CI: 0.542–0.918), indicating moderate predictive performance.

**Conclusion:**

Abnormal BBB permeability is an independent risk factor for structural abnormalities on brain MRI and is positively correlated with the extent of MRI abnormalities. CSF OCB positivity is a risk factor for disease relapse. These findings may inform brain injury assessment, relapse risk stratification, and individualized treatment in patients with anti-NMDAR encephalitis.

## Introduction

Anti-NMDAR encephalitis accounts for approximately 54–80% of autoimmune encephalitis (AE) cases and represents the most common type of AE (Ren et al., 2021). Distinct from classical limbic encephalitis, it aligns with diffuse encephalitis (Wang et al., 2022) and exhibits the most diverse clinical manifestations. Reports indicate that 30–55% of patients with anti-NMDAR encephalitis present with abnormal brain MRI findings (Dalmau et al., 2008; Khatib et al., 2025), while approximately 25% demonstrate abnormal signals in medial temporal lobe structures of the limbic system (including the hippocampus and amygdala) (Dalmau et al., 2008). Additionally, 23–30% of patients show involvement of extralimbic regions such as cortical or subcortical areas, basal ganglia, brainstem, or cerebellum (Dalmau et al., 2019, 2011; Zhang et al., 2018). Multiple studies suggest that abnormal brain MRI findings in anti-NMDAR encephalitis may be associated with poor clinical outcomes and serve as a predictor of adverse prognosis (Khatib et al., 2025; Zhang et al., 2018; Zhao et al., 2022).

Emerging evidence has attempted to link these conventional MRI lesions to underlying BBB impairment, yet inconsistent results exist across different detection modalities. A small-sample dynamic contrast-enhanced MRI study (Ji et al., 2024) revealed universal elevated BBB permeability in all enrolled anti-NMDAR encephalitis patients, with more severe leakage within FLAIR abnormal lesions, implying nearly 100% BBB dysfunction in MRI-abnormal individuals, whereas CSF QAlb-based research reported an overall BBB disruption rate of merely 15.6% without grouping by MRI results (Gong et al., 2023). Regional BBB permeability was correlated with treatment response (Ji et al., 2024). Thus, it remains unclear whether BBB damage represents the predominant component of abnormal conventional MRI findings.

Currently, numerous studies have investigated the relationship between BBB permeability and the pathogenesis as well as prognosis of various central nervous system disorders, such as multiple sclerosis (MS) (Balasa et al., 2021; Jasiak-Zatońska et al., 2022), neuromyelitis optica spectrum disorders (NMOSD) (Jasiak-Zatońska et al., 2022), anti-NMDAR encephalitis (Yu et al., 2021) and herpes simplex encephalitis (Zhang et al., 2025). Abnormal BBB permeability serves as a critical pathophysiological basis for central nervous system (CNS) inflammation, leading to plasma protein leakage, immune cell infiltration, and further amplification of local immune responses. OCB testing relies on paired CSF-serum specimens to distinguish intrathecal from systemic immunoglobulin production. OCB positivity indicates intrathecal immunoglobulin synthesis, which is a key biomarker of disease activity. Meanwhile, brain MRI, as a routine clinical evaluation tool, can reveal varying lesion patterns involving the limbic system, cortex, and multiple brain regions. However, whether the severity of these abnormalities can indirectly reflect BBB dysfunction and relapse risk remains unexplored in systematic studies. In other words, the specific associations of CNS inflammation activation, BBB integrity disruption, and structural brain involvement with clinical symptoms and relapse risk remain unclear.

This study aims to explore the association of intracranial MRI lesions from all brain regions (without predefined restriction) and BBB permeability with clinical symptoms and long-term relapse risk in Chinese patients with anti-NMDAR encephalitis, providing evidence-based support for individualized treatment.

## Materials and methods

### Study population

**Figure 1:**
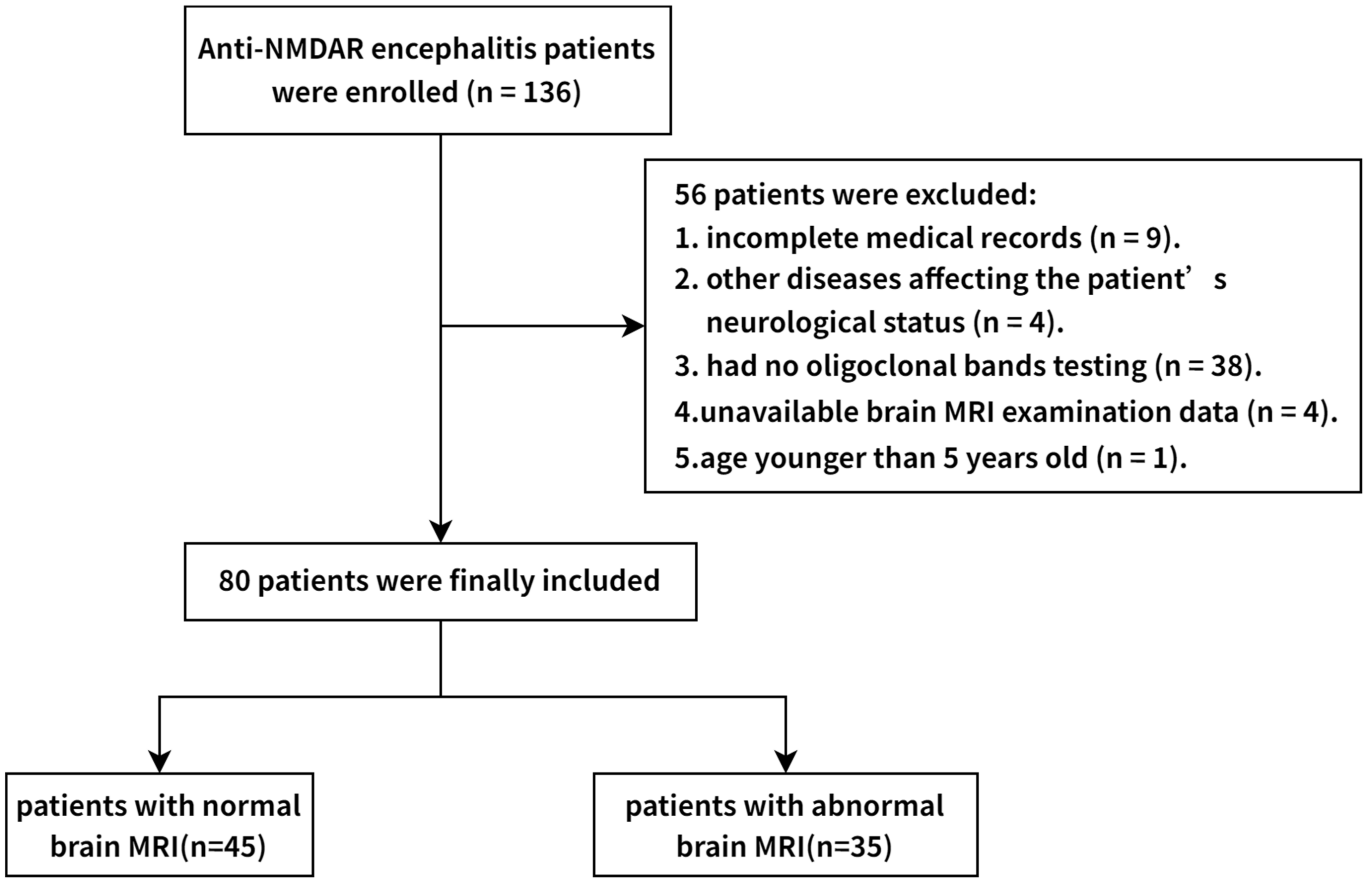
Flow chart of study patients. NMDAR, N-methyl-D-aspartate receptor.

As shown in Figure 1, this retrospective cohort study included 80 patients diagnosed with anti-NMDAR encephalitis at the First Affiliated Hospital of Guangxi Medical University between June 2017 and January 2024, all of whom met the 2016 International Diagnostic Criteria for AE (Graus et al., 2016). To evaluate the potential impact of brain MRI on disease progression, patients were divided into an abnormal MRI group (*n* = 35) and a normal MRI group (*n* = 45). The study was approved by the Ethics Committee of the First Affiliated Hospital of Guangxi Medical University (2026-E0351). All participants or their legal guardians signed written informed consent forms. All patient data were anonymized and used solely for scientific research analysis, in strict adherence to the ethical principles of the Declaration of Helsinki.

### Data collection

Our study systematically collected essential clinical data from medical records. These included demographic characteristics (e.g., age and gender) and clinical features (such as prodromal symptoms, memory impairment, cognitive dysfunction, speech disorders, psychiatric and behavioral disturbances, epileptic seizures, altered consciousness, motor dysfunction, and autonomic dysfunction). Additional data comprised hospitalization duration, intensive care unit (ICU) admission status, laboratory test results (including CSF and peripheral blood analyses), relapse history, and detailed information on immunotherapy.

OCB detection was performed via immunoblotting after isoelectric focusing. All tests were outsourced to a certified clinical laboratory with commercial kits; anti-human IgG antibodies were used to detect CSF IgG. Positivity was strictly defined as the presence of one or more discrete IgG bands in CSF that were absent in corresponding serum samples. Qualitative results (positive/negative) were used for analysis. Disruption of the BBB was determined from the CSF and blood albumin level ratio (Qalb) (Hillmer et al., 2022). As Qalb is age dependent (Seeliger et al., 2024), the individualized age-related Qalb was determined as Qalb* = (4 + Age/15) × 10^–3^ (Reiber, 1994). In the current study, abnormal BBB permeability in adults was defined as Qalb > Qalb*. The original NMDAR antibody titers were expressed as 1:X ratios (e.g., 1:10). During statistical analysis, these titers were converted into numerical variables X. The intrathecal IgG synthesis rate was calculated using the Tourtellotte formula, with positivity defined as > 3.3 mg/day (Tourtellotte et al., 1980). Disease severity and prognosis were assessed using the mRS and Clinical Assessment Scale for Autoimmune Encephalitis (CASE). The mRS was collected as a continuous variable ranging from 0 to 6: score 0 = no symptoms, 1 = minor symptoms without disability, 2 = slight disability, 3 = moderate disability, 4 = moderately severe disability, 5 = severe disability, 6 = death. Peak mRS was defined as the maximum mRS value recorded during the acute hospitalization of anti-NMDAR encephalitis. The CASE score consists of 9 items (seizure, memory dysfunction, psychiatric symptoms, consciousness, language problems, dyskinesia/dystonia, gait instability and ataxia, brainstem dysfunction, weakness), each scored from 0 to 3, yielding a total score ranging from 0 to 27, with higher scores indicating more severe encephalitis (Lim et al., 2019). To ensure comprehensive follow-up, all patients underwent monitoring for at least 12 months after discharge. Relapse was defined as symptom reappearance in AE patients after symptom improvement or stabilization lasting more than 2 months, or symptom exacerbation (defined as an increase of ≥1 point on the mRS score).

### MRI acquisition and analysis

Brain MRI was obtained during the first documented acute attack, including T1-weighted images, T2-weighted images, and T2-FLAIR images. All MRI scans were anonymized and randomized. Two independent neurologists, who were blinded to all clinical information and the original radiology reports, independently reviewed the entire set of scans. Any disagreement between the two readers was resolved by consensus discussion. If consensus could not be reached, a third senior neuroradiologist made the final decision. Inter-rater reliability was assessed and is reported in the Statistical analysis section.

Based on the imaging grading criteria of previous relevant studies (Wang et al., 2022; Zhang et al., 2018; Feng et al., 2022; Gombolay et al., 2023; Wardlaw et al., 2013), intracranial MRI abnormalities were categorized into three grades according to the extent and severity of brain parenchymal involvement: Grade 0 (no abnormalities): no abnormal signals observed; Grade 1 (mild focal abnormalities): localized abnormalities in a single unilateral brain region without significant edema or multi-region involvement; Grade 2 (widespread/severe abnormalities): bilateral limbic system involvement, simultaneous involvement of ≥ 2 brain regions, or extensive lesions accompanied by marked parenchymal edema.

Typical vs. nonspecific lesions: Typical lesions were defined as T2/FLAIR hyperintensities involving the limbic system (unilaterally or bilaterally) or other brain regions (e.g., cortex, basal ganglia, thalamus, brainstem), excluding nonspecific white matter changes and stroke. Nonspecific lesions were defined as isolated punctate or small patchy T2/FLAIR hyperintensities in the periventricular, deep, or subcortical white matter (i.e., White Matter Hyperintensities), without concomitant limbic-system or cortical inflammatory FLAIR changes.

### Statistical analysis

Statistical analysis was performed using IBM SPSS 27.0 software, and figures were created using GraphPad Prism 11. Inter-rater reliability for the three-grade brain MRI abnormality severity scale (0 = no abnormalities, 1 = mild focal abnormalities, 2 = widespread/severe abnormalities) was assessed using Cohen’s kappa (*κ*). Values > 0.80 indicated excellent agreement. To minimize overfitting given the expected limited number of relapse events, we applied the events per variable (EPV) principle (recommended EPV ≥ 5) and constructed a directed acyclic graph (DAG) to guide variable selection in multivariable analysis.

Continuous data were expressed as mean ± standard deviation (mean ± SD) if normally distributed, with intergroup comparisons conducted using the independent samples *t*-test. For non-normally distributed data, median (interquartile range) [M (P25, P75)] was used, and intergroup comparisons were performed using the Mann–Whitney *U* test. Categorical variables were presented as counts (percentages) [*n* (%)], with intergroup comparisons analyzed using the *χ*^2^ test; Fisher’s exact test was employed when expected frequencies were < 5. Correlation analysis utilized the nonparametric Spearman’s rank correlation. Candidate variables with *p* < 0.05 in univariate analyses were included in the multivariable regression model. Furthermore, ROC curve analysis was used to evaluate the predictive efficacy. Calibration of the logistic regression model was assessed using the Hosmer–Lemeshow goodness-of-fit test. A calibration plot was generated to visually compare predicted probabilities with observed relapse frequencies across deciles of predicted risk. A *p*-value < 0.05 was considered statistically significant.

## Results

### Clinical characteristics of study participants

This study included 80 patients with anti-NMDAR encephalitis, comprising 44 females and 36 males. Among them, 45 patients (56.25%) exhibited normal brain MRI findings, while 35 patients (43.75%) showed abnormal brain MRI results. The demographic and clinical characteristics of both groups are presented in Table 1.

**Table 1:** Demographic and clinical characteristics of anti-NMDAR encephalitis patients.

Variable	Total (*n* = 80)	Normal brain MRI (*n* = 45)	Abnormal brain MRI (*n* = 35)	*p-*value
Gender, *n* (%)				0.910
Male	36 (45.0)	20 (44.4)	16 (45.7)	
Female	44 (55.0)	25 (55.6)	19 (54.3)	
Age, median (P25, P75), years	18.5 (15.0, 27.5)	17 (15, 28)	21 (15, 41)	0.186
Clinical manifestations, *n* (%)				
Prodromal symptoms	34 (42.5)	17 (37.8)	17 (48.6)	0.333
Memory deterioration	22 (27.5)	10 (22.2)	12 (34.3)	0.231
Cognitive impairment	55 (68.8)	31 (68.9)	24 (68.6)	0.976
Speech dysfunction	47 (58.8)	30 (66.7)	17 (48.6)	0.103
Mental behavior disorder	71 (88.8)	40 (88.9)	31 (88.6)	0.964
Epileptic seizure	56 (70.0)	32 (71.1)	24 (68.6)	0.806
Disturbance of consciousness	35 (43.8)	17 (37.8)	18 (51.4)	0.222
Movement disorders	51 (63.8)	27 (60.0)	24 (68.6)	0.429
Autonomic nervous dysfunction	36 (45.0)	18 (40.0)	18 (51.4)	0.308
Admission mRS score	3 (3, 4)	3 (2.5, 4)	3 (3, 4)	0.490
Admission CASE score	6 (4, 12)	5 (4,12)	7 (4,12)	0.612
ICU admission, *n* (%)	18 (22.5)	9 (20.0)	9 (25.7)	0.544
Immunotherapy				
Time to first line immunotherapy initiation, median (P25, P75), days	12.00 (8.75, 28.25)	12 (9, 21)	25 (9, 38)	**0.017**
Time to second line immunotherapy initiation, median (P25, P75), days	30 (18, 47.75)	30 (17, 41)	29 (19, 56)	0.673
Hospital stays, median (P25, P75), days	15.00 (11.00, 21.00)	15 (10, 21)	14 (9, 23)	0.835

NMDAR, N-methyl-D-aspartate receptor; mRS, modified Rankin scale; CASE, clinical assessment scale for autoimmune encephalitis; ICU, intensive care unit. Bold values indicate statistical significance (*P* < 0.05).

A significant difference was observed between patients with normal and abnormal brain MRI findings in the time from onset to first-line immunotherapy. Compared to those with normal brain MRI, patients with abnormal brain MRI exhibited a significantly prolonged time from onset to first-line immunotherapy (*p* = 0.017), indicating delayed initiation of first-line immunotherapy in the abnormal MRI group. No significant differences were found in baseline characteristics such as gender, age, clinical symptoms (e.g., memory deterioration, cognitive impairment), ICU admission duration, or admission mRS and CASE scores (all *p-*values > 0.05).

Teratoma was identified in only 3 patients (3.75%). Notably, all 3 had negative MRI findings at baseline and remained relapse-free during the first year of follow-up.

### Characteristics of brain MRI lesions

Of the 35 patients with abnormal brain MRI findings, 21 had multiple lesions. Twenty-six patients (32.5%) presented with typical anti-NMDAR encephalitis abnormalities, including hippocampal lesions (*n* = 7, 8.75%), temporal lobe lesions (non-hippocampal) (*n* = 12, 15.0%), frontal lobe lesions (*n* = 17, 21.25%), insular lobe lesions (*n* = 3, 3.75%), and cingulate gyrus lesions (*n* = 2, 2.5%). Six patients (7.5%) demonstrated nonspecific lesions. Of the 41 patients who underwent contrast-enhanced MRI, enhancement was observed in 5 patients. Among the 26 patients with typical brain lesions, the hippocampal lesion subgroup (*n* = 7, male:female = 3:4) and non-hippocampal lesion subgroup (*n* = 19, male:female = 9:10) showed no significant difference in gender distribution (Fisher’s exact test, *p* > 0.05). See Table 2 for details.

**Table 2:** Brain findings of anti-NMDAR encephalitis patients.

Brain MRI findings	Cases (*n* = 80)	Ratio (%)
Normal	45	56.25
Abnormal	35	43.75
Multiple lesions	21	26.25
Enhancement brain lesions	5	6.25
Hippocampus	7	8.75
Temporal lobe	12	15.0
Frontal lobe	17	21.25
Insular lobe	3	3.75
Cingulate gyrus	2	2.5
Non-specific lesions	6	7.5

NMDAR, N-methyl-D-aspartate receptor.

### Relationship between abnormal BBB permeability and brain lesions

**Figure 2:**
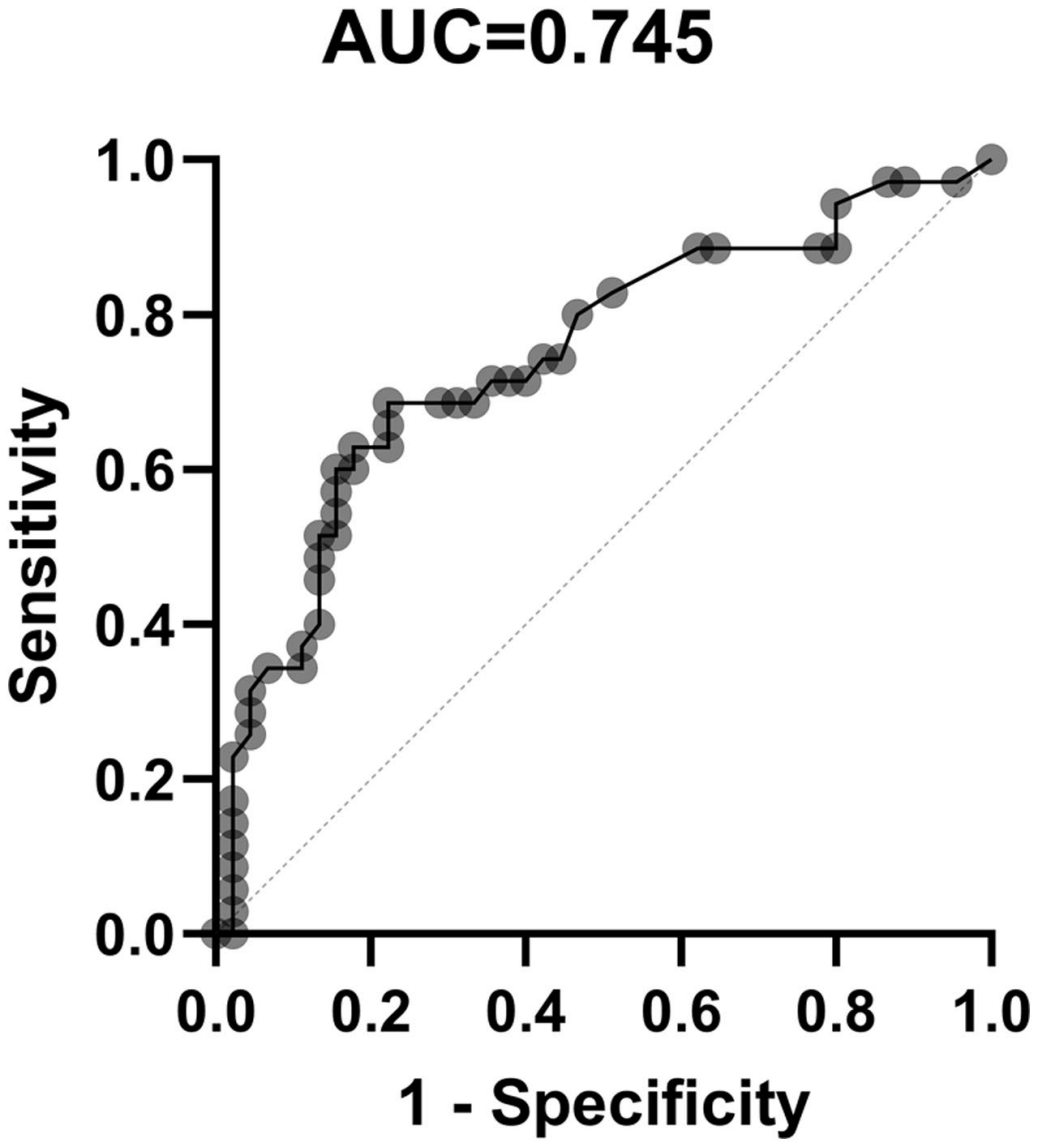
Receiver operating characteristic (ROC) curve assessing the predictive value of BBB dysfunction and time from onset to first-line immunotherapy for abnormal brain MRI lesions in patients with anti-NMDAR encephalitis, with an area under the curve (AUC) of 0.745 (95% CI: 0.634–0.857). AUC, area under the curve.

Laboratory test data are summarized in Table 3. In patients with anti-NMDAR antibodies, Qalb was significantly higher in the abnormal brain MRI group than in the normal MRI group (*p* = 0.009). Total CSF protein levels were also significantly higher in the abnormal brain MRI group (*p* = 0.011). The proportion of patients with abnormal BBB permeability (Qalb > Qalb*) in the abnormal brain MRI group was significantly higher than in the normal group (42.9% vs. 13.3%, *p* = 0.003). The difference in abnormal 24-h intrathecal IgG synthesis rate was also statistically significant (68.6% vs. 28.9%, *p* < 0.001). Abnormal BBB permeability and abnormal 24-h intrathecal IgG synthesis rate were significantly elevated in the abnormal brain MRI group, suggesting that BBB disruption and intrathecal immune activation may be closely associated with abnormal brain MRI findings. In addition, as shown in Table 4, BBB dysfunction (OR = 4.088, 95% CI: 1.031–16.213, *p* = 0.045) and time from onset to first-line immunotherapy (OR = 1.041, 95% CI: 1.001–1.083, *p* = 0.045) were identified as predictive factors for abnormal brain MRI findings. The predictive value of the combined model of BBB dysfunction and time from onset to first-line immunotherapy for brain MRI abnormalities was evaluated by ROC curve analysis, with an area under the curve (AUC) of 0.745 (95% CI: 0.634–0.857), as presented in Figure 2.

**Table 3:** Summary of associations of baseline cerebrospinal fluid measures with brain MRI lesions.

CSF findings	Normal brain MRI (*n* = 45)	Abnormal brain MRI (*n* = 35)	*p-*value
Qalb (×10^−3^)	3.46 (2.84, 4.13)	4.79 (2.70, 6.79)	**0.009**
Abnormal BBB permeability (Qalb > Qalb*)	13.3% (6/45)	42.9% (15/35)	**0.003**
CSF WBC cell count, 10^6^/L	8.00 (2.00, 26.50)	10.00 (2.00, 68.00)	0.354
CSF total protein, mg/L	271.20 (184.60, 360.90)	350.70 (176.40, 550.70)	**0.011**
OCB positivity	48.9% (22/45)	57.1% (20/35)	0.463
Elevated 24-h IgG-index (>3.3 mg/day)	28.9% (13/45)	68.6% (24/35)	**<0.001**
NMDAR antibody titer, median (P25, P75)			
CSF NMDAR antibody titers	30 (10, 32)	10 (10, 32)	**0.039**
Serum NMDAR antibody titers	10 (10, 66)	10 (10, 100)	0.360
Time from symptom onset to LP (days), median (P25, P75)	13.0 (8.0–25.0)	22.0 (7.0–31.0)	0.423
Time from symptom onset to MRI (days), median (P25, P75)	16.0 (7.5–31.5)	20.0 (9.0–34.0)	0.506

NMDAR, N-methyl-D-aspartate receptor; Qalb, albumin-CSF/serum-quotient; OCB positivity, oligoclonal band positivity. Bold values indicate statistical significance (*P* < 0.05).

**Table 4:** Binary logistic regression analysis of correlation between brain MRI and baseline clinical characteristics.

Baseline characteristics	*B*	SE	OR (95% CI)	*p*-value
Onset age	0.005	0.019	1.005 (0.969–1.043)	0.780
Abnormal BBB permeability (Qalb > Qalb*)	1.408	0.703	4.088 (1.031–16.213)	**0.045**
24-h IgG index elevation (>3.3 mg/day)	1.005	0.578	2.732 (0.880–8.485)	0.082
Cerebrospinal fluid NMDAR antibody titer	−0.007	0.005	0.993 (0.983–1.004)	0.207
time to initiation of first-line immunotherapy	0.040	0.020	1.041 (1.001–1.083)	**0.045**

NMDAR, N-methyl-D-aspartate receptor; Qalb, albumin-CSF/serum-quotient. Bold values indicate statistical significance (*P* < 0.05).

Notably, the CSF NMDAR antibody titers in patients with normal brain MRI were significantly higher than those in patients with abnormal brain MRI findings (*p* = 0.039). Nevertheless, multivariate logistic regression analysis indicated that CSF NMDAR antibody titer was not an independent risk factor for brain MRI abnormalities. No significant differences were observed between the two groups in CSF white blood cell count, OCB positivity, or serum NMDAR antibody titers.

### Relationship between abnormal BBB permeability and distribution of brain MRI lesions

Further investigation into the relationship between BBB permeability and brain MRI lesions revealed significant differences in the distribution of lesion sites between the BBB-normal and BBB-abnormal groups. The proportion of patients with no abnormal brain MRI findings was significantly higher in the BBB-normal group (66.1% vs. 28.6%, *p* = 0.001), whereas the proportion of mixed-affected sites was significantly higher in the BBB-abnormal group (33.3% vs. 6.8%, *p* = 0.002). No statistically significant differences were observed in the distribution of purely limbic system involvement, purely other brain parenchyma involvement, or purely meningeal involvement between the two groups (all *p* > 0.05). Of the 41 patients who underwent contrast-enhanced MRI, 5 showed enhancement (enhancement-positive group), including 2 with abnormal BBB permeability (40.0%); the remaining 36 showed no enhancement (enhancement-negative group), including 7 with abnormal BBB permeability (19.4%). No statistically significant difference was found in the frequency of contrast enhancement on brain MRI between the BBB-normal group and the BBB-abnormal group (*p* = 0.299) (Table 5).

**Table 5:** Analysis of correlation between BBB permeability and distribution of brain MRI lesions.

Brain MRI lesions	Normal BBB permeability (Qalb ≤ Qalb*)	Abnormal BBB permeability (Qalb > Qalb*)	*p*-value
No abnormalities, *n* (%)	39 (66.1)	6 (28.6)	**0.001**
Limbic system (hippocampus, amygdala, insular lobe, cingulate gyrus), *n* (%)	5 (8.5)	2 (9.5)	0.702
Meninges, *n* (%)	0 (0.0)	1 (4.8)	0.263
Involvement of other cortical regions (parietal lobe, occipital lobe), *n* (%)	11 (18.6)	5 (23.8)	0.592
Multiregional involvement (≥2 anatomical regions), *n* (%)	4 (6.8)	7 (33.3)	**0.002**
Contrast-enhanced brain lesions, *n* (%)	3 (9.4%)	2 (22.2%)	0.299

Bold values indicate statistical significance (*P* < 0.05).

**Figure 3:**
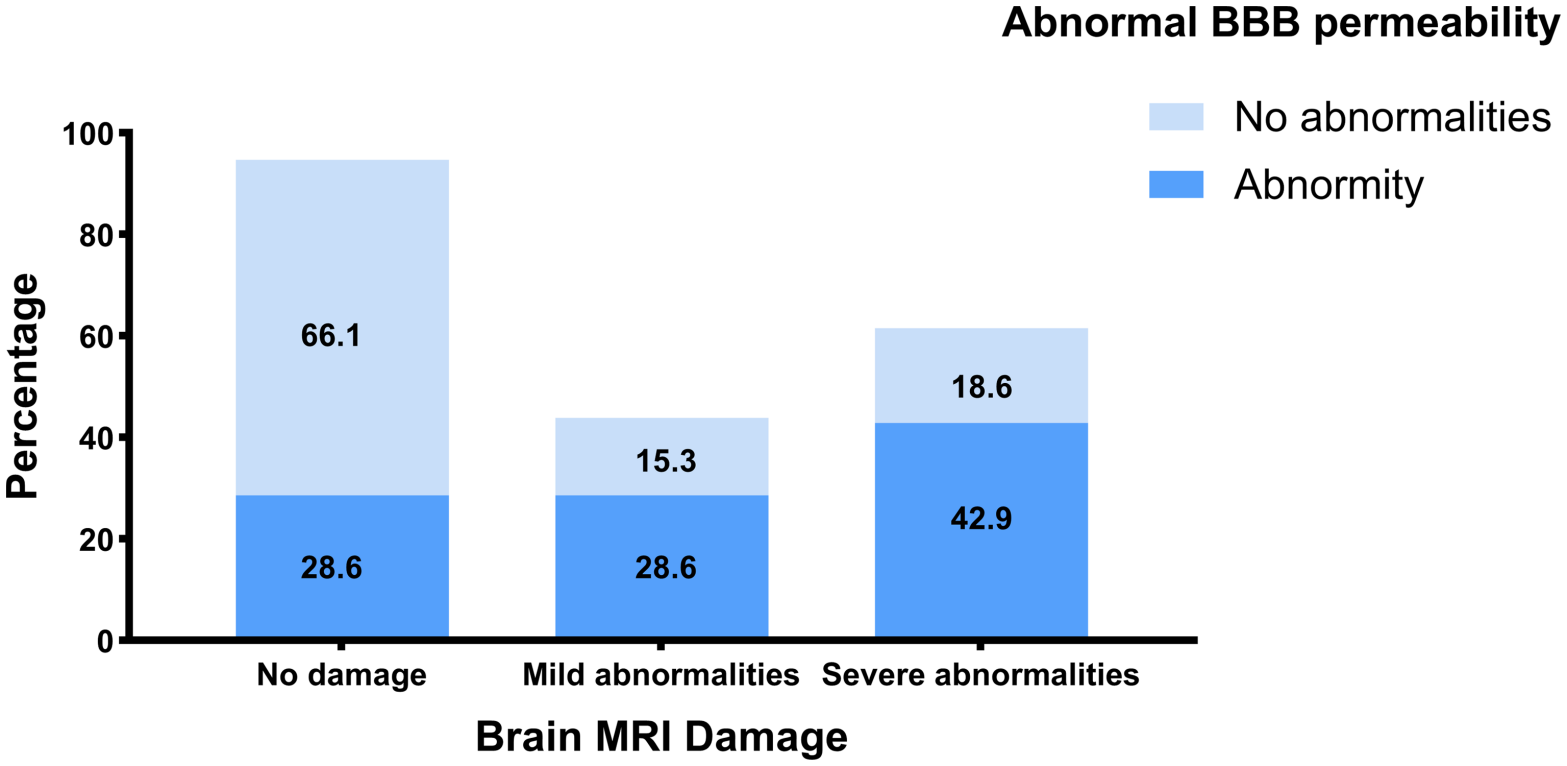
Abnormal blood–brain barrier permeability rates at different injury levels on brain MRI in patients with anti-NMDAR receptor antibodies.

As shown in Figure 3, the BBB permeability rates increased with the severity of intracranial MRI abnormalities in patients with anti-NMDAR encephalitis. Inter-rater reliability analysis included all 80 patients. Cohen’s kappa was 0.850 (SE = 0.052, *p* < 0.001), demonstrating excellent agreement and good reproducibility of the MRI abnormality severity grading. Spearman rank correlation revealed a significant positive correlation between abnormal BBB permeability and MRI lesion severity (Rho = 0.330, *p* = 0.003), indicating that impaired BBB integrity corresponds to more severe brain lesions and broader lesion distribution.

### Predictive factors for relapse in patients with anti-NMDAR encephalitis

Eighty patients were stratified into relapse-free (*n* = 69) and relapse (*n* = 11) groups according to post-discharge relapse status. Table 6 compares the clinical manifestations between the two groups. The results showed statistically significant differences between the two groups in Qalb, OCB positivity, CSF NMDAR antibody titer and serum NMDAR antibody titer (*p* < 0.05). No significant differences were observed between the two groups in gender, age, clinical manifestations, abnormal BBB permeability, CSF white blood cell count, CSF total protein, abnormal MRI findings, time from onset to initiation of first-line immunotherapy, application of second-line immunotherapy, or peak mRS score (all *p* > 0.05).

**Table 6:** Comparison of clinical characteristics between relapse-free and relapse groups in anti-NMDAR encephalitis.

Variable	Total (*n* = 80)	Relapse-free (*n* = 69)	Relapse (*n* = 11)	*p-*value
Gender, *n* (%)				1.000
Male	36 (45.0%)	31 (44.9%)	5 (45.5%)	
Female	44 (55.0%)	38 (55.1%)	6 (54.5%)	
Age, median (P25, P75), years	18.50 (15.00, 27.50)	18.00 (15.00, 29.50)	20.00 (15.50, 24.50)	0.417
Clinical manifestations, *n* (%)				
Prodromal symptoms	34 (42.5%)	27 (39.1%)	7 (63.6%)	0.189
Memory deterioration	22 (27.5%)	17 (24.6%)	5 (45.5%)	0.164
Cognitive impairment	55 (68.8%)	48 (69.6%)	7 (63.6%)	0.733
Speech dysfunction	47 (58.8%)	42 (60.9%)	5 (45.5%)	0.347
Mental behavior disorder	71 (88.8%)	60 (87.0%)	11 (100.0%)	0.347
Epileptic seizure	56 (70.0%)	49 (71.0%)	7 (63.6%)	0.726
Disturbance of consciousness	35 (43.8%)	30 (43.5%)	5 (45.5%)	1.000
Movement disorders	51 (63.8%)	44 (63.8%)	7 (63.6%)	1.000
Autonomic nervous dysfunction	36 (45.0%)	30 (43.5%)	6 (54.5%)	0.531
Hospital stays, median (P25, P75), days	15.00 (11.00, 21.00)	16.00 (11.50, 22.00)	11.00 (6.00, 18.50)	0.148
ICU admission, *n* (%)	18 (22.5%)	16 (23.2%)	2 (18.2%)	1.000
Auxiliary examination results				
Qalb (×10^−3^)	3.76 (2.70, 5.87)	3.38 (2.41, 5.19)	5.56 (4.04, 7.06)	**0.023**
Abnormal BBB permeability (Qalb > Qalb*)	21 (26.3%)	16 (23.2%)	5 (45.5%)	0.146
CSF WBC cell count, 10^6^/L	8.00 (2.00, 22.75)	8.00 (2.00, 25.00)	2.00 (0.00, 18.00)	0.579
CSF total protein, mg/L	285.05 (219.60, 444.78)	283.60 (208.65, 425.00)	290.90 (178.00, 510.00)	0.304
OCB positivity	42 (52.5%)	33 (47.8%)	9 (81.8%)	**0.036**
Elevated 24 h IgG-index (>3.3 mg/day)	37 (46.3%)	30 (43.5%)	7 (63.6%)	0.213
CSF NMDAR antibody titers	30 (10, 32)	10 (10, 32)	32 (30, 32)	**0.037**
Serum NMDAR antibody titer	10 (10, 83)	10 (10, 32)	32 (10, 100)	**0.041**
MRI abnormalities, *n* (%)	35 (43.8%)	29 (42.0%)	6 (54.5%)	0.521
MRI enhancement, *n* (%)	5 (6.3%)	4 (5.8%)	1 (9.1%)	0.776
Immunotherapy				
Time to first line immunotherapy initiation, median (P25, P75), days	12.00 (8.75, 28.25)	12.00 (8.50, 31.50)	10.00 (9.00, 18.50)	0.175
Whether to initiate secondary immunotherapy	46 (57.5%)	37 (53.6%)	9 (81.8%)	0.106
peak mRS score	3 (3,4)	3 (3,4.5)	3 (3,4)	0.832
Peak CASE score	7 (4,13)	7 (4, 14)	8 (4, 10)	0.844

ICU, intensive care unit; Qalb, albumin-CSF/serum-quotient; OCB positivity, oligoclonal band positivity; NMDAR, N-methyl-D-aspartate receptor; mRS, modified Rankin scale; CASE, clinical assessment scale for autoimmune encephalitis. Bold values indicate statistical significance (*P* < 0.05).

**Figure 4:**
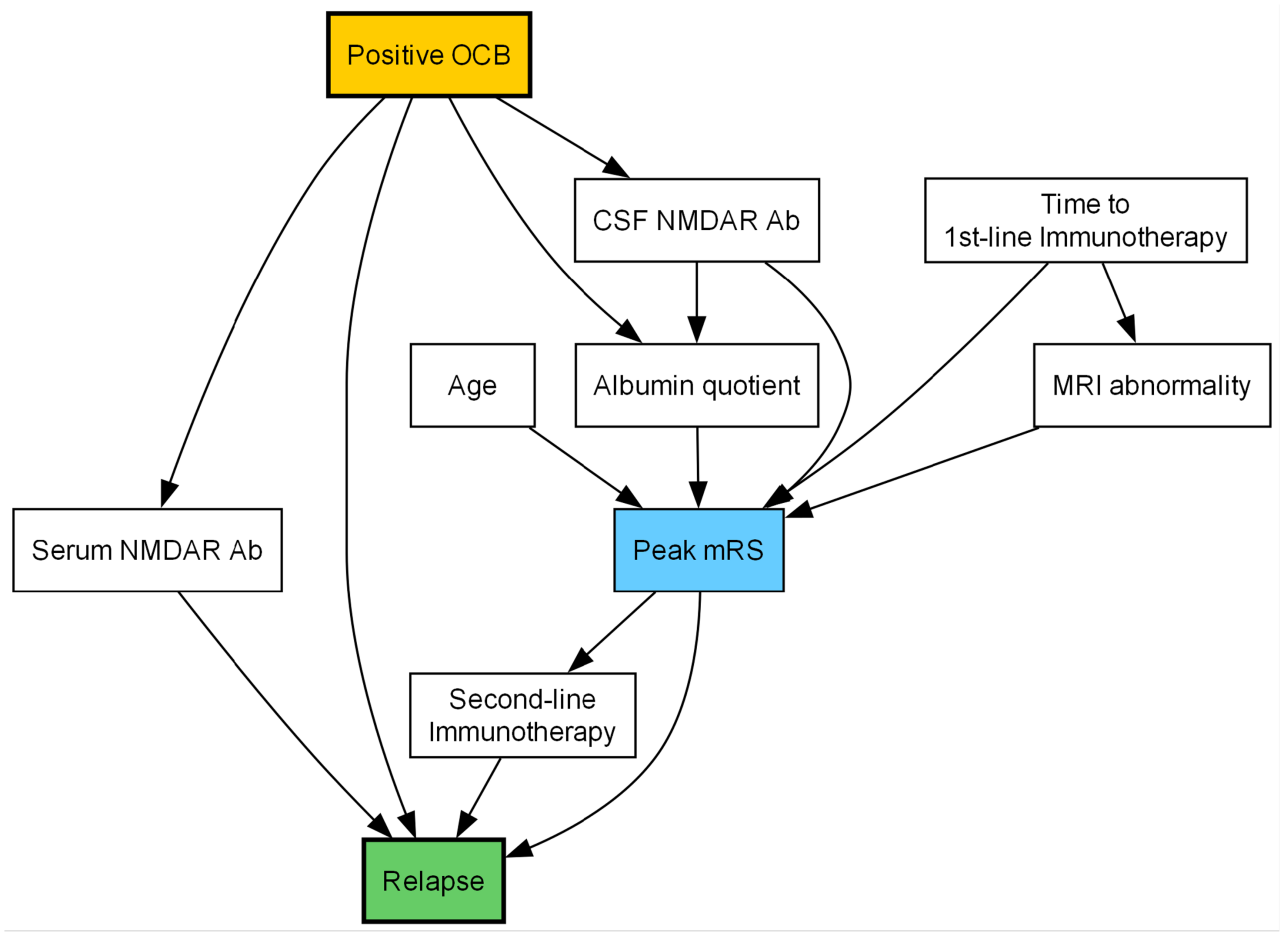
Directed acyclic graph (DAG) guiding variable selection for the relapse analysis. OCB, oligoclonal band; NMDAR, N-methyl-D-aspartate receptor; mRS, modified Rankin scale; CSF, cerebrospinal fluid; Ab, antibody. Yellow: exposure (OCB); Blue: covariate (mRS); Green: outcome (relapse); White: unadjusted variables. Arrows indicate causal directions.

Variables with statistical significance (*p* < 0.05) in univariate analysis were included in the multivariate logistic regression model to explore the predictive factors for relapse of anti-NMDAR encephalitis. Considering the limited number of relapse cases (*n* = 11), and based on the EPV principle, we further applied a DAG to guide variable selection while minimizing overfitting. As shown in Figure 4, the DAG specified the causal relationships among all candidate variables. The DAG identified peak mRS as a confounder located on the backdoor path between OCB positivity and relapse. According to the backdoor criterion, adjusting for peak mRS alone is sufficient to estimate the total effect of OCB on relapse. Other univariably significant variables (Qalb, CSF/serum antibody titers) were identified as mediators on the causal pathway and were therefore excluded to avoid overadjustment bias. Variables without univariable significance (age, time from onset to first-line immunotherapy, MRI abnormalities, and second-line immunotherapy) were also excluded, as they either showed no association with relapse or their effects were mediated through peak mRS.

Consequently, the final parsimonious multivariable model included only OCB positivity as the core predictor and peak mRS score as a covariate to adjust for confounding by disease severity, yielding an EPV ratio of 5.5 (11 events per 2 variables). This falls below the conventional threshold of 10 but is within the range reported in previous exploratory studies of rare events. Therefore, these findings should be interpreted as hypothesis-generating and require validation in larger cohorts.

**Figure 5:**
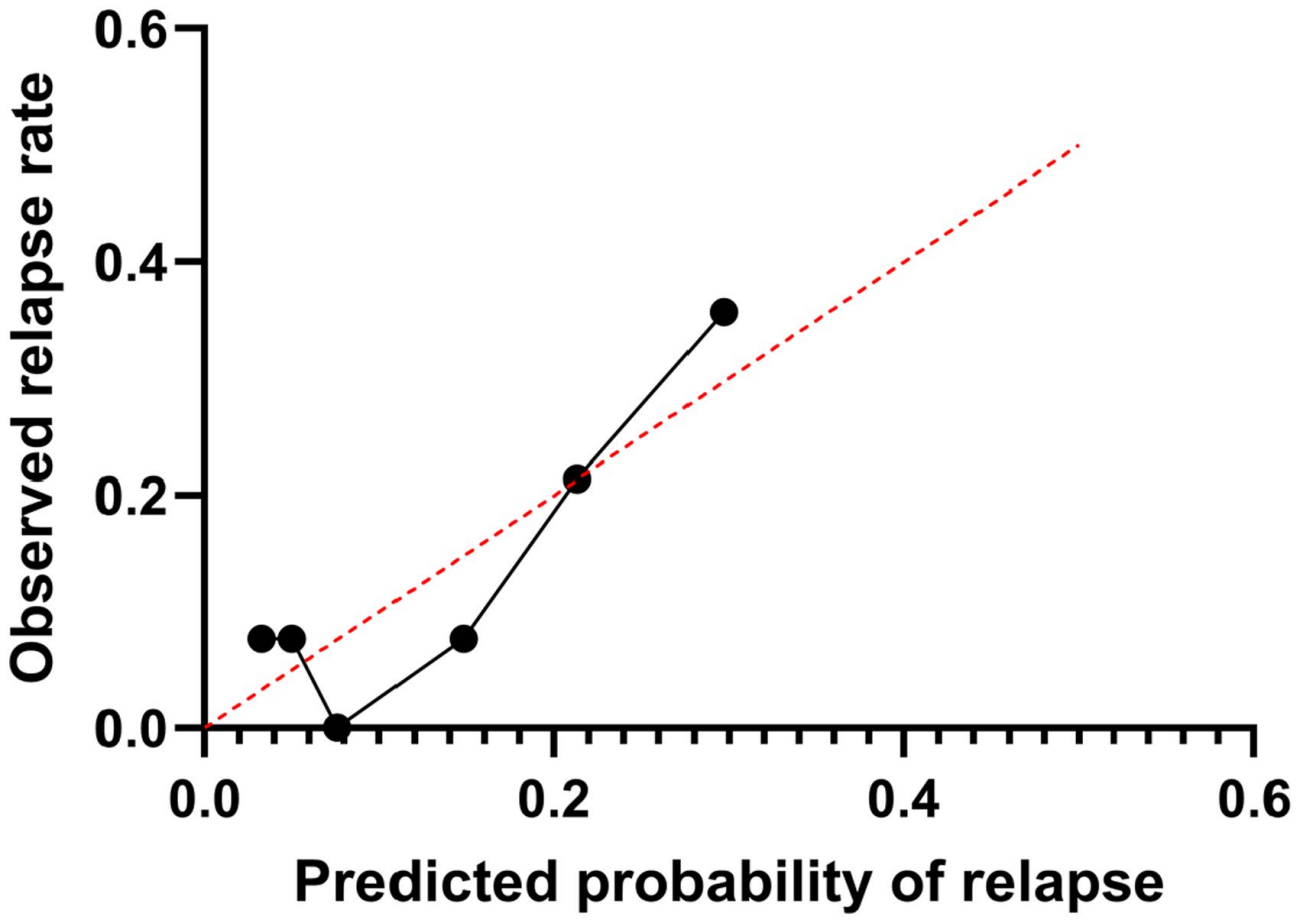
Calibration curve. Dashed diagonal line: ideal calibration. Solid line: observed relapse rates. The model showed acceptable calibration, with minor overestimation at the highest risk stratum.

**Figure 6:**
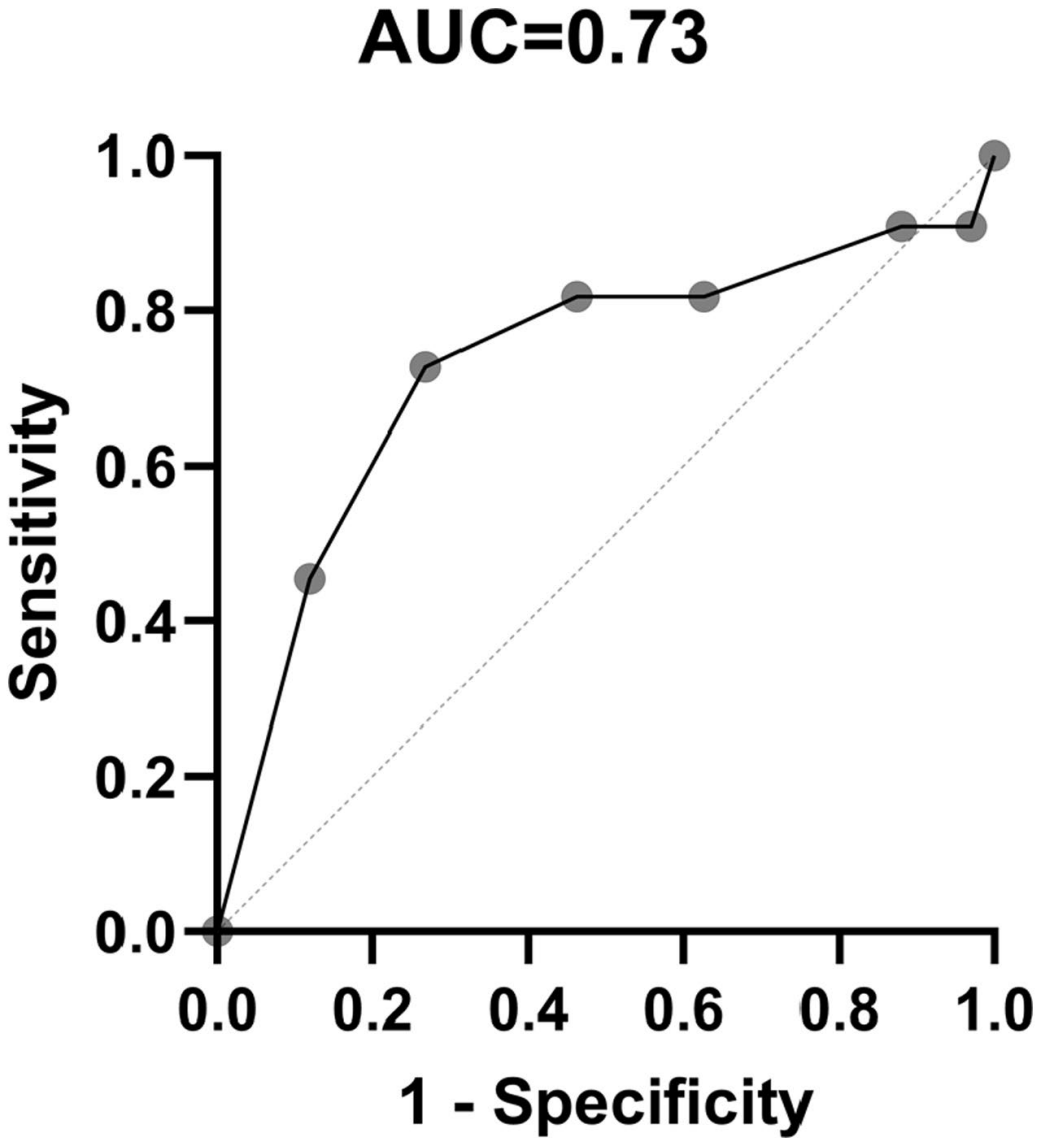
ROC curve analysis of OCB positivity for predicting anti-NMDAR encephalitis relapse. AUC, area under the curve.

The logistic regression model included OCB positivity and mRS score as predictors of relapse. The Hosmer–Lemeshow test was not statistically significant (*χ*^2^ = 4.510, df = 4, *p* = 0.341), indicating no evidence of poor calibration. This was further supported by the calibration plot (Figure 5), which showed generally good agreement between predicted probabilities and observed relapse rates along the diagonal line. However, the plot also revealed a slight deviation at the highest predicted risk level (>0.40), where the observed relapse rate was marginally lower than predicted. After adjusting for the confounding effect of mRS score, OCB positivity remained significantly associated with anti-NMDAR encephalitis relapse (OR = 7.991, 95% CI: 1.242–51.411, *p* = 0.029), as presented in Table 7. As shown in Figure 6, ROC curve analysis demonstrated that the combined model (OCB positivity + mRS score) had a moderate predictive efficacy for relapse, with an area under the curve (AUC) of 0.730 (95% CI: 0.542–0.918).

**Table 7:** Multivariate regression analysis: predictors of relapse in patients with anti-NMDAR encephalitis.

Variable	*B*	SE	OR (95% CI)	*p*-value
Peak mRS score	−0.443	0.416	0.642 (0.284–1.452)	0.287
OCB positivity	2.078	0.950	7.991 (1.242–51.411)	**0.029**

NMDAR, N-methyl-D-aspartate receptor; OCB positivity, oligoclonal band positivity.

## Discussion

The BBB acts as the primary structural barrier separating the brain from the systemic circulation. By regulating molecular trafficking between the CNS and the bloodstream, the BBB exerts a critical immunoprotective role for the CNS. Anti-NMDAR encephalitis is an antibody-mediated autoimmune encephalitis characterized by the presence of anti-NMDAR antibodies in the CSF. Activation of NMDARs can induce disruption of the BBB (Yu et al., 2022) and enhance BBB permeability (Yu et al., 2022; Mao et al., 2022). In the CNS, NMDARs are widely distributed, not only in the cortex and hippocampus but also in regions such as the amygdala, striatum, nucleus accumbens, cerebellum, brainstem, spinal cord, and peripheral nervous system (Beaurain et al., 2024). Abnormal brain MRI findings in anti-NMDAR encephalitis predominantly manifest as involvement of the limbic system, specifically the NMDAR-enriched regions of the hippocampus and amygdala (Dalmau et al., 2008; Khatib et al., 2025). However, whether these abnormal MRI manifestations are directly associated with compromised BBB integrity remains to be further verified.

This study systematically investigated the correlations among BBB function, intrathecal immune status, and brain MRI lesions in patients with anti-NMDAR encephalitis. Patients in the abnormal brain MRI group had significantly higher Qalb levels (*p* = 0.009) and a higher incidence of abnormal BBB permeability (*p* = 0.003) compared with the normal MRI group, suggesting that BBB disruption is more severe in patients with abnormal MRI findings. The CSF total protein level was also significantly elevated in the abnormal MRI group (*p* = 0.011), further suggesting that impaired BBB function may lead to the infiltration of peripheral protein components into the CNS. The proportion of patients with an abnormal 24-h intrathecal IgG synthesis rate was significantly increased (68.6% vs. 28.9%, *p* < 0.001), indicating that intrathecal immune activation was more prominent in the abnormal MRI group. Patients with abnormal brain MRI may suffer from concurrent severe BBB disruption and augmented intrathecal immunoglobulin synthesis, both of which may jointly participate in the pathological process of brain parenchymal injury.

Notably, this study also found that CSF NMDAR antibody titers in patients with normal brain MRI were significantly higher than those in patients with abnormal MRI findings (*p* = 0.039). Although binary logistic regression showed that CSF NMDAR antibody titer was associated with abnormal brain MRI findings (OR = 0.993, *p* = 0.207) rather than serving as an independent causal predictor, this inverse correlation indicated that the association between NMDAR antibody levels and brain MRI abnormalities was not a simple linear relationship. This finding may suggest that patients with higher antibody titers present with more active immune responses in the acute phase, whereas obvious structural brain lesions have not yet developed. This finding is highly consistent with the pathophysiological characteristics of anti-NMDAR encephalitis. Anti-NMDAR antibodies mainly downregulate receptor function via synaptic immune responses, presenting with reversible neurological impairment as the core clinical manifestation (Dalmau et al., 2011; Hughes et al., 2010).

To explore this hypothesis, we compared the time from symptom onset to LP between the two MRI groups. The median time was 13.0 days (IQR 8.0–25.0) in the normal MRI group versus 22.0 days (IQR 7.0–31.0) in the abnormal MRI group, indicating a trend toward earlier sampling in the normal group. Although this difference did not reach statistical significance (Mann–Whitney *U* = 705.0, *p* = 0.423)—likely due to the wide distribution in the abnormal group and limited sample size—the directional consistency supports the interpretation that higher antibody titers in patients with normal MRI reflect early immune activation rather than established structural damage. Thus, high antibody titers tend to reflect early immune activation rather than structural brain damage. This finding may inform clinical risk stratification. For patients with high CSF antibody titers but normal MRI, early immune activation should be monitored closely, and immunotherapy should be initiated promptly to prevent progression to structural lesions. Large-sample, multicenter prospective studies are required to further verify these results and explore the correlation between dynamic changes in antibody titers and clinical prognosis.

This study confirmed that abnormal BBB permeability is a strong independent predictor of abnormal brain MRI findings (OR = 4.088, *p* = 0.045), indicating that disruption of BBB integrity is not merely an incidental manifestation but a core pathological process driving cerebral MRI abnormalities. Impairment of BBB integrity serves as an important marker of CNS inflammation involving the brain parenchyma, suggesting that immune inflammation has crossed the barrier and induced structural alterations including cerebral edema and glial activation, consequently presenting as abnormal signals on MRI. In contrast, elevated CSF antibody titers alone merely reflect intrathecal immune activation. In the absence of BBB damage, structural lesions rarely occur and MRI remains normal. Meanwhile, delayed first-line immunotherapy is associated with a significantly increased risk of abnormal MRI findings (*p* = 0.045). This finding suggests that timely immune intervention is crucial for optimizing MRI outcomes, which has important clinical implications for diagnosis and treatment.

Inconsistent with previous studies, hippocampal abnormalities accounted for only 8.75% of patients with abnormal brain MRI findings, frontal lobe abnormalities for 21.25%, and multiple lesions accounted for 26.25%. Abnormal brain MRI manifestations in anti-NMDAR encephalitis patients were mostly characterized by multi-regional brain damage. On this basis, Spearman rank correlation analysis showed a positive correlation between abnormal BBB permeability and the severity of cerebral MRI lesions. Worse BBB permeability was associated with more severe structural brain damage and wider lesion involvement on MRI. The observed trend toward higher BBB permeability in patients with contrast enhancement did not reach statistical significance (*p* = 0.299), possibly due to the small sample size and limited statistical power of this subgroup analysis. These findings suggest that clinical assessment of BBB function can be used as an important indicator for evaluating the severity of brain parenchymal injury in anti-NMDAR encephalitis. Patients with BBB disruption should receive intensive immune intervention as early as possible to alleviate structural brain damage and improve clinical prognosis.

Anti-NMDAR encephalitis is the most common type of AE in clinical practice. Most patients achieve a favorable prognosis after immunotherapy, whereas some experience symptom recurrence or clinical relapse. However, the underlying mechanisms and predictive markers of relapse remain incompletely clarified. To address this gap, we retrospectively enrolled 80 patients with anti-NMDAR encephalitis. They were divided into a relapse group (*n* = 11) and non-relapse group (*n* = 69) according to post-discharge relapse status. The relapse rate was 13.75%. Univariate analysis showed that OCB positivity, CSF NMDAR antibody titer, serum NMDAR antibody titer, and Qalb were statistically associated with relapse (all *p* < 0.05).

The non-significant protective tendency of peak mRS (OR < 1) was explained by confounding by indication. Patients with higher peak mRS routinely received prolonged intensive maintenance immunosuppressive therapy per clinical guideline, which reduced their subsequent relapse risk; in contrast, mild patients with low mRS scores usually did not receive long-term maintenance treatment and thus had a higher baseline relapse risk. However, this trend failed to reach statistical significance, so peak mRS was not an independent relapse predictor.

Given the limited number of relapse events (*n* = 11), correlation analysis was used for variable screening. Finally, the core variable (OCB positivity) and the key confounding factor (mRS score) were included in the regression model to avoid overfitting. After adjusting for the peak mRS score during the acute phase, OCB positivity remained an independent risk factor for relapse of anti-NMDAR encephalitis (OR = 7.991, 95% CI: 1.242–51.411, *p* = 0.029), which is consistent with previous research findings (Abboud et al., 2021). CSF OCB is a classic marker of intrathecal specific immunoglobulin synthesis in the CNS, directly reflecting persistent intracranial immune activation. During the course of AE, persistent OCB positivity generally indicates uncontrolled central immune dysregulation. Residual autoreactive lymphocytes continuously produce pathogenic antibodies, thereby increasing the risk of clinical relapse. Accordingly, patients with OCB positivity should receive intensified follow-up monitoring and prolonged immunomodulatory treatment to reduce the risk of disease relapse.

After adjustment for confounding factors, OCB-positive patients with anti-NMDAR encephalitis had more than a 7-fold higher risk of relapse compared with OCB-negative patients. This suggests that intrathecal immune abnormality is not merely a concomitant manifestation during the acute stage, but a core pathological mechanism driving disease chronicity and relapse. ROC curve analysis yielded an AUC of 0.730 for predicting relapse. This finding may be attributed to the limited sample size and small number of relapse cases, which reduced statistical power; however, this limitation does not negate its clinical predictive value. Compared with serum and CSF NMDAR antibodies, CSF OCB positivity can serve as a biological marker for evaluating relapse risk in anti-NMDAR encephalitis and exhibit more stable and reliable early warning value for relapse.

### Limitations

This study has several limitations. Firstly, the total sample size was relatively small, including only 80 patients with merely 11 relapse cases. Although the Hosmer-Lemeshow test was non-significant (*χ*^2^ = 4.510, df = 4, *p* = 0.341), the calibration curve revealed a modest deviation at the highest risk stratum, which was possibly attributable to the limited sample size in that subgroup. Insufficient data on monitoring indicators may have resulted in low statistical power for the ROC curve analysis. Secondly, as a single-center retrospective study, potential selection bias was inevitable. In addition, dynamic changes in imaging findings and antibody titers during long-term follow-up were not observed, making it impossible to clarify their temporal evolutionary patterns. Third, teratoma status could not be formally stratified due to only 3 cases in our cohort. All 3 had negative MRI and no relapse at 1 year, consistent with our main finding, but larger studies are needed to assess potential interaction. Fourth, LP timing was not standardized (median 14.5 days; range 3–96 days), and we did not adjust for disease duration, which may have introduced confounding. Future studies with standardized sampling are warranted. Finally, detailed long-term intervention information, such as immunological maintenance regimens, treatment duration and drug withdrawal protocols, was not systematically recorded, which may affect disease relapse risk. Owing to the small sample size and single-center retrospective design, our findings require further verification by large-sample, multicenter prospective studies, with emphasis on dynamic antibody alterations and the effects of maintenance immunotherapy on clinical prognosis.

## Conclusion

In summary, our results demonstrate that BBB function assessment can be used to evaluate the risk of brain parenchymal injury in clinical practice. When combined with CSF OCB positivity, this assessment enables relapse risk stratification. Intensive follow-up surveillance and standardized maintenance immunotherapy should be adopted for high-risk patients to alleviate structural brain damage, reduce the relapse rate, and optimize long-term clinical prognosis.

## Data Availability

The original contributions presented in the study are included in the article/supplementary material, further inquiries can be directed to the corresponding author.
